# Common molecular pathways involved in human CD133+/CD34+ progenitor cell expansion and cancer

**DOI:** 10.1186/1475-2867-7-11

**Published:** 2007-06-08

**Authors:** Oswaldo Keith Okamoto, Ana Carolina SR Carvalho, Luciana C Marti, Ricardo Z Vêncio, Carlos A Moreira-Filho

**Affiliations:** 1 Departamento de Neurologia e Neurocirurgia, Universidade Federal de São Paulo – Escola Paulista de Medicina, São Paulo, Brazil; 2 Instituto Israelita de Ensino e Pesquisa Albert Einstein, São Paulo, Brazil; 3 Institute for Systems Biology, Seattle, WA 98103-8904, USA; 4 Departamento de Imunologia, Instituto de Ciências Biomédicas, Universidade de São Paulo, São Paulo, Brazil

## Abstract

**Background:**

Uncovering the molecular mechanism underlying expansion of hematopoietic stem and progenitor cells is critical to extend current therapeutic applications and to understand how its deregulation relates to leukemia. The characterization of genes commonly relevant to stem/progenitor cell expansion and tumor development should facilitate the identification of novel therapeutic targets in cancer.

**Methods:**

CD34+/CD133+ progenitor cells were purified from human umbilical cord blood and expanded *in vitro*. Correlated molecular changes were analyzed by gene expression profiling using microarrays covering up to 55,000 transcripts. Genes regulated during progenitor cell expansion were identified and functionally classified. Aberrant expression of such genes in cancer was indicated by *in silico *SAGE. Differential expression of selected genes was assessed by real-time PCR in hematopoietic cells from chronic myeloid leukemia patients and healthy individuals.

**Results:**

Several genes and signaling pathways not previously associated with *ex vivo *expansion of CD133+/CD34+ cells were identified, most of which associated with cancer. Regulation of MEK/ERK and Hedgehog signaling genes in addition to numerous proto-oncogenes was detected during conditions of enhanced progenitor cell expansion. Quantitative real-time PCR analysis confirmed down-regulation of several newly described cancer-associated genes in CD133+/CD34+ cells, including *DOCK4 *and *SPARCL1 *tumor suppressors, and parallel results were verified when comparing their expression in cells from chronic myeloid leukemia patients

**Conclusion:**

Our findings reveal potential molecular targets for oncogenic transformation in CD133+/CD34+ cells and strengthen the link between deregulation of stem/progenitor cell expansion and the malignant process.

## Background

Hematopoiesis is a tightly regulated process with important clinical and therapeutic implications. Understanding how expansion of hematopoietic stem and progenitor cells is regulated may reveal molecular mechanisms associated with leukemogenesis. Another practical goal of this knowledge is to devise techniques for expanding the amount of such progenitors from human umbilical cord blood (UCB), in sufficient levels for allogeneic transplants in adults suffering from hematological malignancies [1,2].

The CD133 antigen has been considered as a new marker of primitive hematopoietic stem cells (HSC) capable of supporting hematopoiesis both *in vitro *and *in vivo *[3,4]. Circulating stem cells in UCB characterized by co-expression of CD34 and CD133 markers display a certain degree of plasticity, which expands their prospective therapeutic use [5]. This subpopulation of CD133+/CD34+ cells also shows higher engraftment in NOD/SCID mice than CD133-/CD34+ cells [6] and clinical-scale isolation of functional CD133+ cells can be performed from cryopreserved UCB samples [7].

Several recent studies have focused on conditions for *ex vivo *expansion of CD133+ cells [8-10]. Nonetheless, few studies have addressed potential biosafety concerns such as chronic effects and tumorigenesis. Indeed, tumors may arise from the neoplastic transformation of normal stem cells, supported by the fact that some signaling pathways known to be involved in stem cell self-renewal and proliferation of progenitors are also associated with cancer [11]. Although expression profiling studies have been conducted comparing CD133+ with their CD133- counterparts in attempt to identify "stemness"-related genes [12-15], the molecular events associated with expansion of these primitive progenitors is still poorly described.

Mutations in normal somatic stem and progenitor cells leading to deregulation of their physiological programs is thought to increase their predisposition to tumor development. Under this cancer stem cell hypothesis viewpoint, the identification of genes commonly relevant to stem/progenitor cell and tumor biology should facilitate the development of novel therapeutic approaches in cancer.

In this work, we report conditions for basal and high yield *in vitro *expansion of a CD133+/CD34+ subset of progenitor cells from human UCB stimulated by estradiol, a hormone involved in both normal cell proliferation and development of cancer. A detailed molecular characterization of such processes is provided from a genomic perspective. Overlapping groups of genes and signaling pathways involved in both progenitor expansion and cancer development were identified, including newly described tumor suppressor genes of great therapeutic interest.

## Results

### Expansion of CD133+ and CD34+ cells from UCB

**Figure 1:**
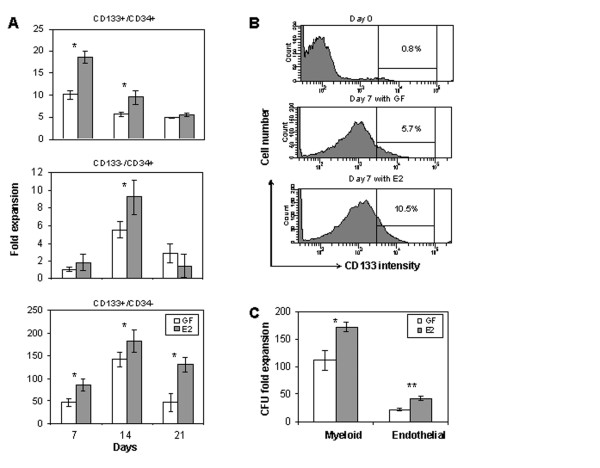
*In vitro *expansion of stem and progenitor cells from UCB cultivated in the presence or absence of estradiol. (A) Fold increment in CD133+/CD34+, CD133-/CD34+, and CD133+/CD34- cell number after 7, 14, and 21 days in culture. (B) Flow cytometry analysis showing percentage of mononucleated cells expressing CD133 prior to and after culturing for 7 days. Positive and isotype controls are provided in supplemental figure S2. (C) Functional clonogenic assay based on the frequency of colony forming units (CFU). Data correspond to fold expansion of myeloid and endothelial progenitors at culture day 7. GF = basal medium supplemented with growth factors only (SCF, IL3, IL6, and Flt3-ligand); E2 = medium supplemented with growth factors plus 10 nM estradiol. Statistical significance for GF vs. E2 comparisons: * *P *< 0.01, **P < 0.001.

Flow cytometry analysis revealed that most CD34+ cells (82 ± 13%) also express CD133, a primitive human HSC marker [see Additional file 1]. Upon cultivation in a defined medium supplemented with growth factors (SCF, IL3, IL6, and Flt3-ligand), the absolute amount of CD133+ and CD34+ cells was estimated after 7, 14 and 21 days. Highest increment in CD133+/CD34+ cell number occurred at day 7 (10.0 ± 1.1 fold). The amount of more committed CD133-/CD34+ cells also increased, with a maximum of 5.5 ± 0.9 fold observed at day 14. At the same culture day, a robust increment (141.9 ± 16.5 fold) in primitive CD133+/CD34- stem cell subset was detected (Fig. 1A).

Under the same time-frame, higher expansion of these three cell populations was obtained when cells were treated with 10 nM E2. Total amounts of CD133+/CD34+ (at day 7), CD133-/CD34+ (at day 14) and CD133+/CD34- (at day 14) cells were 18.7 ± 1.4, 9.2 ± 1.9, and 181.8 ± 20.4 fold higher in the presence of E2, respectively, than at day 1. The CD133+ cells are rare primitive stem cells usually representing less then 1% of the total mononuclear cell fraction. In addition to increased cell number, higher proportion of CD133+ cells were verified in 7-day cultures, particularly in the presence of E2 (Fig. 1B).

In order to determine whether these UCB stem cells retained their function after expansion, their clonogenic activity was examined *in vitro*. The amount of CFU of myeloid and endothelial type was increased by a factor of 100 and 20, respectively, after seven days in basal growth medium (SCF, IL3, IL6, and Flt3-ligand only). Such increment was enhanced nearly two fold after addition of 10 nM E2 (Fig.1C).

### Genes involved in abrogation of CD133+/CD34+ cell quiescence

With the purpose of defining the pool of transcripts active in UCB CD133+/CD34+ stem cells, we performed a microarray hybridization using a platform containing more than 55,000 oligonucleotide probes representing all annotated human genes to date. Description of the bioarrays used is constantly updated by the manufacturer at the GEO database under the accession numbers GPL2895 and GPL2891. The starting total RNA used in the microarray hybridizations was extracted from freshly isolated, non-cultivated, CD133+/CD34+ cells, immediately after their purification from UCB. A typical microarray-based analysis of gene expression was applied in order to clarify the molecular events associated with their *ex vivo *expansion, under basal growth conditions.

A total of 851 up-regulated genes and 991 down-regulated genes were found, as a consequence of expansion for seven days in basal growth medium (day-7 vs. day-0 comparison). A myriad of biological processes and molecular functions could be related to these differentially expressed genes [see Additional file 1]. However, the overall representation of genes and corresponding annotated functions in the entire human genome may confer a bias when grouping genes based on functional similarity. Furthermore, a single gene may belong to multiple categories. Therefore, a statistical test was applied to find functional categories over-represented in the lists of differentially expressed genes, in attempt to distinguish processes and functions specifically associated with cell expansion from those present due to random chance.

**Figure 2:**
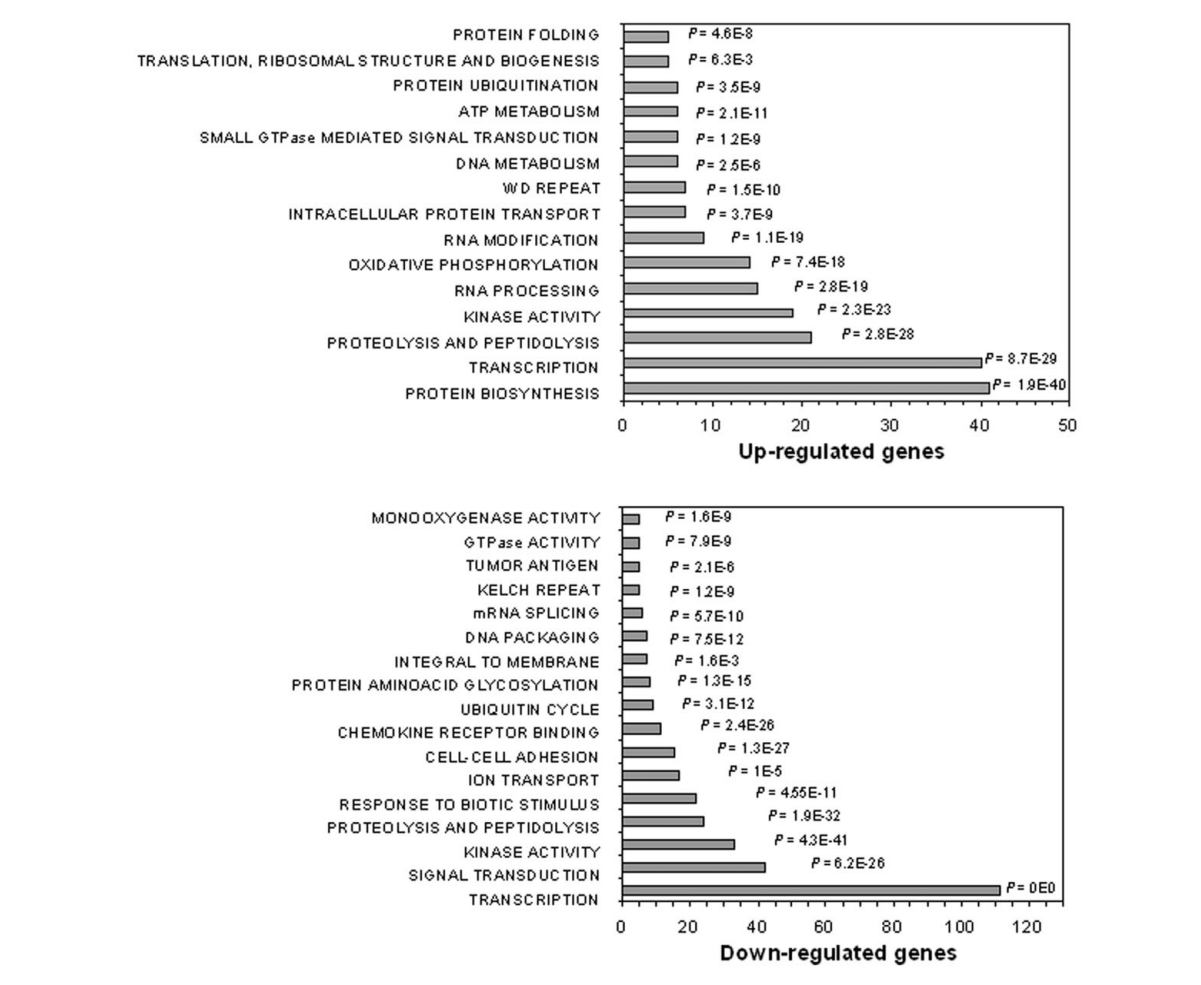
Gene-Enrichment and Functional Annotation Analysis. Main functional categories of differentially expressed genes associated with expansion of CD133+/CD34+ cells in basal medium supplemented with growth factors (SCF, IL3, IL6, and Flt3-ligand). Statistical significance: *P*-values = 0.001 for all comparisons, according to the Fisher Exact Test for ontology enrichment analysis.

Such classification strategy revealed that the list of up-regulated genes is specially enriched in genes functioning in primary metabolism (e.g. RNA synthesis and processing, protein metabolism, and ATP biosynthesis), while transcription regulation and signal transduction are over-represented among the down-regulated genes. Figure 2 summarizes the number of enriched genes, their corresponding molecular functions, and respective *P*-values for functional association, indicating that expansion of CD133+/CD34+ cells involves an intense metabolic activity and regulation of gene expression at all levels. The list of differentially expressed genes classified by enriched functional category is provided [see Additional file 2].

The largest enriched functional group consisted of over 100 transcription factors that were down-regulated during the basal growth condition tested. Most of them code for zinc finger (41%) and homeobox (20%) proteins, with many functioning in development control. The up-regulated group of genes was also enriched in transcription factors, mainly zinc fingers (45%) and basal transcription factors (10%) required for general transcription.

### Enhanced CD133+/CD34+ cell expansion correlates with regulation of genes abnormally expressed in cancer

Since superior *ex vivo *expansion of CD133+/CD34+ cells was observed when cultivated in the presence of E2, the next step aimed at identifying the early on genes and regulatory pathways possibly accounting for such effect. The gene enrichment and functional classification analysis of a set of 394 genes showing greater than two fold differences in expression after 1h-treatment with 10 nM E2 (222 up- and 172 down-regulated), revealed that transcription regulation, protein modification/degradation, and signal transduction are over-represented classes in both up- and down-regulated gene lists (Table 1). In the transcription factor category, zinc fingers persisted as majority (60% of down-regulated and 39% of up-regulated genes). Almost half of the up-regulated zinc fingers are transcription repressors containing the CRAB domain, suggesting a tight control of gene expression.

**Table 1:** Genes differentially expressed in CD133+/CD34+ cells after 1h-treatment with 10 nM estradiol. Only those genes and their corresponding functional classes found significantly associated with the estradiol treatment are shown (*P*-values = 0.001, according to the Fisher Exact Test for enrichment analysis). Expression data is given as fold change from control, with negative values indicating repression. Additional information on deregulation in cancer and functional evidence of role in tumorigenesis is provided based on *in silico *SAGE and literature datamining*, respectively.

**ACCESSION No.**	**FOLD CHANGE**	**DEREGULATED IN CANCER**	**FUNCTION IN TUMORIGENESIS**	**DESCRIPTION**
				***UP-REGULATED GENES***

**TRANSCRIPTION(*P*-value = 1.3E-17)**				
NM_003426	**2,3**	yes	ND	zinc finger protein 74 (Cos52)
AK024438	**2.1**	yes	ND	hypothetical protein FLJ38705
NM_005419	**2.3**	yes	yes	signal transducer and activator of transcription 2, 113 kDa
NM_032329	**2.5**	yes	yes	inhibitor of growth family, member 5
NM_005239	**2.3**	yes	yes	v-ets erythroblastosis virus E26 oncogene homolog 2 (avian) (ETS2)
NM_032433	**2.1**	yes	ND	zinc finger protein 333
AK024763	**2.1**	yes	ND	small nuclear RNA activating complex, polypeptide 5, 19 kDa
NM_006732	**2.2**	yes	yes	FBJ murine osteosarcoma viral oncogene homolog B (FOSB)
AK024514	**2.6**	yes	yes	suppressor of zeste 12 homolog (Drosophila)
NM_000901	**2.6**	yes	ND	nuclear receptor subfamily 3, group C, member 2
NM_000209	**2.1**	yes	yes	insulin promoter factor 1, homeodomain transcription factor
NM_003073	**2.3**	yes	yes	SWI/SNF related, matrix associated, actin dependent regulator of chromatin, subfamily b, member 1 (SMARCB1)
NM_022465	**2.0**	yes	ND	zinc finger protein, subfamily 1A, 4 (Eos)
NM_006509	**2.0**	yes	yes	v-rel avian reticuloendotheliosis viral oncogene homolog (RELB)
BC032246	**2.1**	yes	ND	zinc finger protein 44 (KOX 7)
AK021493	**2.2**	yes	ND	cAMP responsive element binding protein 5
AF052094	**2.7**	yes	ND	endothelial PAS domain protein 1
AK025453	**2.0**	yes	yes	prospero-related homeobox 1
BI861540	**2.2**	yes	ND	PBX/knotted 1 homeobox 2
NM_005384	**2.7**	yes	ND	nuclear factor, interleukin 3 regulated
NM_145165	**2.2**	yes	ND	churchill domain containing 1
NM_144690	**2.2**	no	ND	zinc finger protein 582
NM_006713	**2.6**	yes	ND	SUB1 homolog (S. cerevisiae)
				
**PROTEIN MODIFICATION (*P*-value = 1.44E-7)**				
AF038042	**2.1**	yes	yes	BRCA1 associated RING domain 1
NM_007218	**2.2**	yes	yes	ring finger protein 139
NM_024686	**2.4**	yes	ND	tubulin tyrosine ligase-like family, member 7
AB011116	**2.2**	yes	ND	mahogunin, ring finger 1
AF070538	**2.5**	yes	ND	tripartite motif-containing 3
NM_024787	**2.9**	yes	ND	ring finger protein 122
AF176705	**2.2**	yes	ND	F-box protein 10
NM_023079	**2.0**	yes	ND	Ubiquitin-conjugating enzyme E2Z (putative)
				
**G-PROTEIN COUPLED RECEPTOR(*P*-value = 3.57E-8)**				
NM_002386	**2.3**	yes	ND	melanocortin 1 receptor (alpha melanocyte stimulating hormone receptor)
NM_002980	**2.8**	yes	ND	secretin receptor
AF303373	**2.1**	no	ND	olfactory receptor, family 4, subfamily D, member 2
NM_005314	**2.4**	yes	ND	gastrin-releasing peptide receptor
NM_030774	**2.0**	no	ND	olfactory receptor, family 51, subfamily E, member 2
NM_004248	**2.2**	no	ND	G protein-coupled receptor 10
M76676	**2.3**	yes	ND	G protein-coupled receptor 135

				***DOWN-REGULATED GENES***

**TRANSCRIPTION (*P*-value = 2.44E-7)**				
NM_030381	**-2.7**	yes	yes	GLI-Kruppel family member GLI2
NM_005920	**-2.2**	yes	yes	MADS box transcription enhancer factor 2, polypeptide D (myocyte enhancer factor 2D)
AF146694	**-4.4**	yes	ND	PHD finger protein 7
NM_017831	**-2.6**	no	ND	ring finger protein 125
NM_003316	**-2.0**	yes	ND	tetratricopeptide repeat domain 3
NM_004234	**-2.2**	no	ND	zinc finger protein 235
U69127	**-2.5**	yes	ND	far upstream element (FUSE) binding protein 3
NM_002466	**-3.1**	yes	ND	v-myb myeloblastosis viral oncogene homolog (avian)-like 2 (MYBL2)
NM_001166	**-2.1**	yes	yes	baculoviral IAP repeat-containing 2
AA001334	**-2.7**	yes	ND	zinc fingers and homeoboxes 2
NM_016166	**-2.2**	yes	ND	protein inhibitor of activated STAT, 1
				
**PROTEOLYSIS AND PEPTIDOLYSIS (*P*-value = 5.75E-9)**				
NM_003343	**-2.0**	yes	ND	ubiquitin-conjugating enzyme E2G 2 (UBC7 homolog, yeast)
NM_023038	**-2.6**	yes	ND	ADAM metallopeptidase domain 19
NM_005800	**-2.5**	yes	ND	Ubiquitin specific peptidase like 1
NM_032582	**-3.6**	yes	ND	ubiquitin specific protease 32
NM_001335	**-2.2**	yes	ND	cathepsin W (lymphopain)
NM_000930	**-2.0**	yes	ND	plasminogen activator, tissue
				
**PROTEIN KINASE CK2 ACTIVITY(*P*-value = 2.1E-10)**				
NM_006482	**-2.8**	yes	ND	dual-specificity tyrosine-(Y)-phosphorylation regulated kinase 2
AF086179	**-2.0**	yes	yes	cyclin-dependent kinase 6
NM_005406	**-2.8**	yes	yes	Rho-associated, coiled-coil containing protein kinase 1
D50683	**-2.0**	yes	yes	transforming growth factor, beta receptor II (70/80 kDa)
NM_003954	**-2.5**	no	ND	mitogen-activated protein kinase kinase kinase 14
NM_014326	**-2.5**	yes	ND	death-associated protein kinase 2

Only those genes and their corresponding functional classes found significantly associated with the estradiol treatment are shown (*P*-values = 0.001, according to the Fisher Exact Test for enrichment analysis). Expression data is given as fold change from control, with negative values indicating repression. Additional information on deregulation in cancer and functional evidence of role in tumorigenesis is provided based on *in silico *SAGE and literature datamining*, respectively.

* Literature data mining was performed in batch by the DAVID 2.1 tool. ND = function not determined at the time of analysis.

One common trait observed for most genes comprising the over-represented functional classes regulated in CD133+/CD34+ cells by E2 is the abnormal expression in tumors. As shown in table 1, from the list of enriched functional categories, 82% of the up-regulated genes and 87% of the down-regulated genes are also deregulated in cancer, several of which having a role in tumorigenesis, according to *in silico *SAGE and literature data mining.

Another strategy used to unraveling processes and functions related with expansion of CD133+/CD34+ stem cells by E2 was to assess the regulatory signaling pathways represented among the differentially expressed genes. Table 2 lists all signaling pathways and corresponding gene members found in such analysis. The data for genes differentially expressed in the E2 treatment or in basal growth medium alone are shown in parallel. From the 10 major regulatory pathways identified, Calcium and MAPK signaling were the ones represented by the largest number of members (> 10). In the MAPK signaling, members of the JNK/p38 MAPK pathway were down-regulated in cells expanded in basal medium and cells under E2 treatment, while members of the MEK/ERK pathway were up-regulated only in E2-treated cells. Members of the Notch and Wnt pathways were regulated solely by the basal expansion condition, while NF-kappaB (represented by *IRAK2 *and *BCL10*) and Sonic Hedgehog (represented by *GLI2*) were found regulated only after E2 treatment.

**Table 2:** Regulatory signal transduction pathways and corresponding gene members found differentially expressed during expansion of CD133+/CD34+ cells.

**Signaling Pathway**	**Control vs. GF**		**GF vs. E2**	
		
	**Up-regulated**	**Down-regulated**	**Up-regulated**	**Down-regulated**
**Calcium**	*GNAQ, GSD VI*	*PDGFB, ADRA1B, ERBA1, CACNA1I, PLCD3, PTK2B*	*CACNA1G, CACNA1D, ADORA2A, GRPR*	*ADCY3, CACNA1I, CACNA1H*
**Hedgehog**	*-*	*-*	*-*	*GLI2*
**Insulin**	*GSD VI*	*IRS4*	*PRKAG3*	*-*
**Jak-STAT**	*PIK3C2B, IL2R, IL5RA*	*CNTF, IL2RG, IFNGR2, IL23A*	*STAT2, CTNF*	*PIAS1, CTF1*
**MAPK**	*-*	*PDGFB, MAPK13, JUN, MEF2C, FGF6*	*RPS6KA1, PLA2G10*	*MAPKAPK3, ARRB1, FLNC, MAP3K14, TGFBR2*
**NF-Kappa B**	*-*	*-*	*-*	*BCL10, IRAK2*
**Notch**	*RBPSUH*	*-*	*-*	*-*
**Phosphatidyl inositol**	*PIK3C2B*	*PLCD3*	*CDS1, DGKE, SYNJ1*	*CSNK1G2*
**TGF-Beta**	*-*	*INHBA, SMAD7*	*E2F4*	*ROCK1, TGFBR2*
**Wnt**	*-*	*DKK4*	*-*	*-*

(Control = freshly isolated cells without cultivation; GF = cells expanded for seven days in basal growth medium; E2 = cells expanded for seven days in basal growth medium plus 1h-treatment with 10 nM estradiol).

### Novel genes involved in CD133+/CD34+ cell expansion with parallel expression pattern in chronic myeloid leukemia

**Figure 3:**
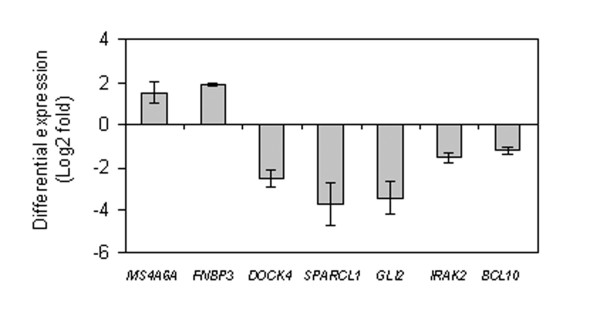
Relative gene expression levels after 1h-treatment with 10 nM estradiol. Transcript levels were quantified by real-time PCR in CD133+/CD34+ cells either with or without estradiol (control). Data are expressed as log2 of fold change. Negative values indicate repression. Statistical significance: *P *< 0.001 for all comparisons.

**Figure 4:**
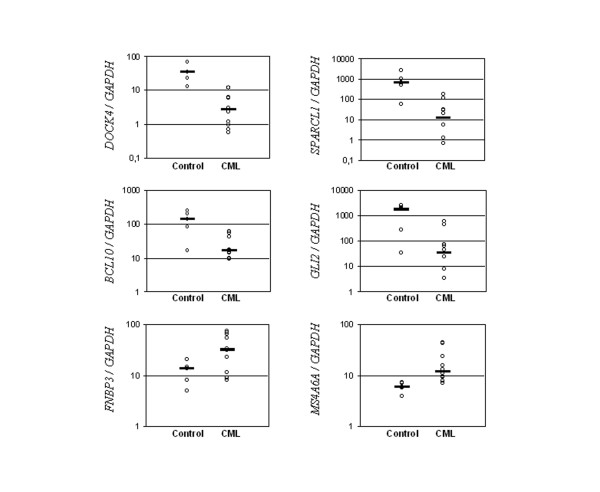
Expression levels of *DOCK4*, *SPARCL1*, *BCL10*, *GLI2, MS4A6A*, and *FNBP3 *genes in healthy individuals (control) and chronic myeloid leukemia patients. Total RNA was extracted from purified mononuclear cells from peripheral blood samples. Transcript levels were quantified by real-time PCR and results are given as normalized gene expression. Horizontal bars indicate median expression values. Statistical significance: *P *< 0.001 for *DOCK4*; *P *< 0.01 for *SPARCL1*, *BCL10*, and *GLI2*; *P *< 0.05 for *MS4A6A *and *FNBP3*.

Several of these cancer-associated genes identified as being regulated during CD133+/CD34+ cell expansion are new and still not fully functionally characterized in the literature, such as *MS4A6A*, *FNBP3*, *DOCK4*, and *SPARCL1*. Differential expression of these genes, in addition to other identified early E2-regulated genes displaying aberrant expression in tumors was confirmed in CD133+/CD34+ cells by quantitative real-time PCR (Fig.3), validating the microarray data. To further examine a possible connection with tumorigenesis, differential expression of the abovementioned genes was examined in the mononuclear cell fraction of healthy volunteers and chronic myeloid leukemia (CML) patients. Significant differences in expression were found for *DOCK4*, *GLI2, BCL10, SPARCL1, MS4A6A*, and *FNBP3 *(Fig.4).

## Discussion

Knowledge of the mechanisms controlling stem/progenitor cell expansion is critical to broaden current therapeutic application for hematopoietic reconstitution and to understand how its deregulation may lead to pathological conditions. To address this issue, we first examined the extent of expansion in a defined medium for distinct stem/progenitor cell subsets. Highest increment in amount was detected for CD133+/CD34- cells, reaching over 100 fold expansion after 14 days. However, most of the CD133+ cells also expressed the membrane protein CD34. This CD133+/CD34+ cell subset accounted for the majority of the cells in culture and exhibited a maximum expansion of 10 fold after seven days. In all cases, addition of E2 in the culture medium resulted in improved expansion and increased amount of clonogenic cells.

Substantial changes in the CD133+/CD34+ cell's transcriptome were detected following expansion in basal growth medium, characterized by differential expression of hundreds of genes. Nearly half of these genes was up-regulated and the other half down-regulated. Using an ontology enrichment analysis, we identified biological processes significantly associated to this expansion condition. There was a striking activation of the cellular primary metabolism, marked by up-regulation of protein and energy biosynthesis, suggesting abrogation of quiescence, a state characteristic of adult stem cells [16]. In agreement with this notion, 82 of the up-regulated genes were identified with a function in cell cycle, including proliferating cell nuclear antigen (PCNA), Cyclins A2 and B1, cell division cycle genes (Cdc2, Cdc20, Cdc25C, and Cdc28), and cyclin-dependent kinases (CDK4, CDK5, and CDK8).

Another evidence for quiescence abrogation is the down-regulation of two members of the growth arrest and DNA damage 45 family, GADD45-beta and GADD45-gamma, known to be activated during stress conditions. These nuclear proteins are ubiquitously expressed in all tissues where they are involved in maintenance of genomic stability, DNA repair, and suppression of cell growth through interaction with cell cycle proteins. Transcriptional silencing of *GADD45*-gamma due to promoter hypermethylation has been recently reported in several tumor cell lines and this epigenetic inactivation correlated with tumorigenesis [17]. In the hematopoietic environment, *GADD45 *deletion has also been shown to support CD4^+ ^T cell proliferation and resistance to apoptosis [18]. Expression of GADD45 genes is known to induce apoptosis via activation of the JNK and p38 MAPK pathway [19]. Accordingly, down-regulation of GADD45-beta and GADD45-gamma in CD133+/CD34+ cells during basal expansion condition was associated with down-regulation of *MAPK13 *(*p38-delta*) as well as of *c-JUN *and *MEF2C*, two target transcription factors of the p38/JNK pathway. The list of down-regulated genes also included 39 genes involved in apoptosis, among which members of the TNF receptor superfamily (TNFRSF9, TNFRSF21, and TNFRSF25) and related proteins (TNFAIP3 and TRAF4), suggesting lower susceptibility to the apoptotic extrinsic pathway.

In connection with the activation of primary metabolism, signal transduction was another biological process significantly associated to the basal expansion of CD133+/CD34+ cells. A specific analysis revealed an intricate modulation and interaction of regulatory signaling pathways, favoring proliferation over differentiation. Under basal growth conditions, the insulin and TGF-beta pathways were mostly down-regulated, implicating in inhibition of glycogen storage and organogenesis. While glycogenesis is linked to energy biosynthesis, inhibition of TGF-beta signaling is coherent with the observed down-regulation of 154 genes involved in development, 89 of them functioning specifically in organogenesis, mostly neurogenesis, hematopoiesis, and general cell differentiation. Furthermore, up-regulation of a gene coding PIK3C2B, a specific isoform of phosphoinositide 3-kinase, indicate stimulation of the AKT/PKB survival signaling, connecting the JAK-STAT and Phosphatidylinositol pathways.

Consistent with the literature, *DKK4*, an inhibitor of the Wnt pathway, was found down-regulated in cells expanded in basal growth condition, supporting the involvement of such pathway in expansion of hematopoietic progenitors [20]. A transcription activator of the CSL family acting downstream the Notch signaling, *RBPSUH*, was also found exclusively up-regulated under the same expansion condition. A recent study with stem/progenitor cells of the small intestine demonstrated that conditional removal of *RBPSUH*, or blockade of the Notch pathway, converts proliferative undifferentiated crypt progenitors into postmitotic goblet cells in a concerted process with the Wnt pathway [21]. More recently, JAG1, a Notch ligand, has been reported to be an evolutionarily conserved target of the Wnt signaling pathway and a mediator of the Wnt-induced self-renewal of HSC derived from embryonic cells [22]. Down-regulation of Notch leads to accelerated differentiation of HSC *in vitro *and depletion of HSC *in vivo*, but does not seem critical for HSC entry into the cell cycle [23].

When CD133+/CD34+ cells were enforced to proliferate by treatment with E2, the major circulating ovarian steroid with known mitogenic effects, up-regulation of *RPS6KA1 *and *PLA2G10*, two members of the MEK/ERK pathway, and down-regulation of *GLI2*, a member of the Hedgehog pathway, were verified. Activation of the Hedgehog signaling pathway has been correlated to maturation of hematopoietic progenitors and neuronal differentiation of mesenchymal stem cells [24,25], whereas constitutive activation of the MEK/ERK pathway stimulates proliferation of erythroid progenitors and blocks their differentiation [26].

Aberrant activation of Wnt, MEK/ERK, and Notch signaling pathways is known to contribute to the pathogenesis of myeloid leukemias and acute T-cell lymphoblastic leukemias and lymphomas [27,28]. Therefore, it seems reasonable to assume that deregulation of stem/progenitor cell expansion is an important factor leading to tumorigenesis. While transient and controlled changes in the expression of specific genes may contribute to stem/progenitor cell maintenance and/or expansion, any event (e.g. mutations, chromosome deletions or duplications) allowing such changes to become permanent could also contribute to neoplastic transformation. In agreement with this concept, a very recently study by Toren *et al*. [15] identified a group of growth factor receptors and transcription factors differentially expressed in CD133+ cells that are mutated in hematological malignancies.

In our model of E2-enhanced expansion of CD133+/CD34+ cells, almost 400 genes displayed immediate response to E2 and are likely involved in the propagation of its biological effect. Although enhanced progenitor cell expansion was detected under E2 treatment, such differentially expressed genes identified in CD133+/CD34+ cells and their corresponding molecular process may also be partially involved with other effects of E2, not addressed in this work. Transcription, post-translational modification and signal transduction were the molecular process significantly associated with the E2 treatment, according to an ontology enrichment analysis. Interestingly, more than 80% of the genes comprising these over-represented functional classes display abnormal expression in several tumor types. While some of these genes have been reported to be involved in tumorigenesis, others are still poorly described in the literature such as *MS4A6A*, which codes for a signal transduction protein of the MS4A family [29], *FNBP3*, a gene encoding another signaling protein of the Rho-related pathway [30], and *IRAK2*, a gene disrupted in HBV-induced hepatocellular carcinomas [31]. Proto-oncogenes typically associated with hematological malignancies, however, were not over-represented.

Down-regulated genes in E2-treated CD133+/CD34+ cells also included the newly characterized tumor suppressor genes *DOCK4*, a member of the CDM gene family, and *SPARCL1*, which encodes an extracellular matrix-associated glycoprotein. Both genes are down-regulated in cancers as a result of missense mutations or chromosomal deletions [32,33]. While *DOCK4 *regulates formation of cellular junctions and is disrupted in prostate and ovarian cancers,*SPARCL1 *acts as a negative regulator of cell proliferation and its down-regulation is detected in prostate, colon, and non-small cell lung carcinomas [32,34,35]. Thus far, differential expression of *DOCK4 *and *SPARCL1 *had never been associated to CD133+/CD34+ cell expansion or leukemias. Notably, when examining *DOCK4 *and *SPARCL1 *expression in CML patients, we found that both genes were also down-regulated compared to healthy individuals, supporting a potential link between deregulation of hematopoietic stem/progenitor cell expansion and the malignant process. Due to their involvement in cancer as a consequence of permanently aberrant expression, pre-clinical studies assessing safety of stem/progenitor cell expansion protocols for therapeutic application are highly demanded. Nevertheless, the potential role of *DOCK4 *and *SPARCL1 *as targets for oncogenic transformation in adult progenitor cells remains to be confirmed.

## Conclusion

In summary, our findings indicate that quiescence abrogation of CD133+/CD34+ cells specifically involves regulation of transcription factors functioning in primary metabolism and development, as well as a concerted activation of genes from the Wnt, AKT/PKB, and Notch signaling pathways. Superior expansion of CD133+/CD34+ cells however, introduces the risk of proto-oncogene regulation. Our E2-enhanced cell expansion model was characterized by the early activation of genes belonging to the MEK/ERK survival pathway and a remarkable regulation of multiple cancer-associated genes. Several genes not previously related to CD133+/CD34+ cell expansion were identified, some of which known to have tumor suppressor activities. Complemented by further studies, we believe that such strategy may help elucidate molecular mechanisms involved in expansion and neoplastic transformation of stem/progenitor cells. Ultimately, new therapeutic interventions in leukemias could be pursued.

## Methods

### Cell purification and culture

Human UCB was collected with informed consent approved by the Internal Review Board of the Albert Einstein Hospital (n = 5). CD133+ and CD34+ cells were purified by immunomagnetic separation with MiniMACS kit (Miltenyi Biotech), following the manufacturer's protocol. Cells were cultivated in Stem Pro medium (Gibco/BRL), supplemented with 2 mM L-glutamine, 50 U/mL penicillin, 50 μg/mL streptomycin, 50 ng/mL Flt-3 ligand, 10 ng/mL interleukin (IL)-3, 10 ng/mL IL-6, and 50 ng/mL stem cell factor, in a humidified atmosphere at 37°C and 5% CO_2_. Cell growth was monitored either with or without addition of 10 nM 17-beta-Estradiol (E2) in the media. This E2 concentration was defined based on previous dose response experiments by *in vitro *assays, as described below.

### *Ex Vivo *Expansion

The amount of CD133+ and CD34+ cells was analyzed on days 0, 7, 14, and 21, by flow cytometry. Clonogenic assays were performed on days 0 and 7. Briefly, for myeloid cell colonies, 5 × 10^3 ^CD133+/CD34+ cells were suspended in 1 mL of semisolid methylcellulose medium supplemented with 10% FCS, 1% bovine serum albumin (BSA), 2 mM L-glutamine, 0.5 mM 2-mercaptoethanol, 10 ng/mL IL-3, 10 ng/ml IL-6, 50 ng/mL stem cell factor, 3 U/mL erythropoietin (StemCell Technologies) and cultivated as above. Total colony forming units (CFUs) were scored microscopically after 14 days (sum of CFU-Mix, BFU-E, and CFU-GM). Endothelial cell colony forming units were analyzed in 1 mL of semisolid methylcellulose medium supplemented with 50 ng/mL VEGF, 50 ng/mL SCF, and 5 ng/mL FGF (Stem Cells Technologies), according to the manufacturer's protocol, and scored as above.

### Flow Cytometry

Cells were incubated with monoclonal antibodies to human antigens CD14 (FITC), CD34 (PE), CD45 (PerCP Cy-5.5), and CD133 (APC; Myltenyi Biotec), with respective isotype controls IgG2a (FITC), IgG1 (PE), IgG1 (PerCP Cy-5.5), and IgG1 (APC) (Becton Dickinson), at 4°C for 30 minutes. Fluorescent cellular events were acquired on the FACSAria flow cytometer and analyzed with FacsDiva software (Becton Dickinson).

### Microarray hybridization

Gene expression profiling was analyzed in freshly isolated CD133+/CD34+ UCB cells prior to cultivation (day 0) and after 7 days in culture either with or without 1h-treatment with 10 nM E2. Independent microarray hybridizations were carried out for each UCB sample, in duplicates, with oligonucleotide microarrays representing approximately 20,000, and 55,000 human transcripts (CodeLink™ Bioarrays, GE Healthcare), following the manufacturer's protocol. Detailed descriptions of these Bioarray platforms are publicly available, according to MIAME guidelines, at the GEO database under the accession numbers GPL2895 and GPL2891. Briefly, total RNA was extracted with RNeasy spin columns and treated with RNAse free-DNAse (Qiagen). One microgram of total RNA was reverse transcribed in the presence of T7- oligo dT primer. The resulting cDNA was used for *in vitro *transcription reaction using T7 RNA polymerase and biotinylated dUTP. Ten micrograms of target cRNA was fragmented at 94°C for 20 min and hybridized to the bioarrays at 37°C for 18 h, under 300 rpm agitation. After staining with streptavidin-conjugated Cyanine-5 dye, the slides were washed and fluorescence was measured using an Axon GenePix 4000B Scanner (Axon Instruments Inc).

### Gene Expression measurement

The fluorescent images were captured using GenePix Pro v.4.1 (Axon Instruments Inc) and the light intensities were quantified, corrected for background noise, and normalized with the CodeLink™ Expression Analysis v4.1. Spots with background contamination, shape irregularity, or pixel saturation were filtered out. Only spots flagged as "good" (G) or "low" (L) were considered in the subsequent analysis. Although the CodeLink™ system is a one-color platform, we grouped the meaningful comparisons in pairs to form ratios, as usual in two-color co-hybridized microarray platforms. The results were analyzed in the MS space, where M = log_2_(expression ratio) and S = log_2_(mean intensity) [36]. Raw data and normalized hybridization data are available according to MIAME guidelines at the GEO database, under the accession number GSE4609.

### Differential expression detection

Differentially expressed genes were identified according to the HTself method [37] which takes advantage of self-self experiments to define an intensity-dependent fold-change cutoff. Since our microarray system is a one-color platform, we built virtual experiments combining two replicates of actual hybridizations. We performed three independent self-self experiments and defined a fold-change cutoff with 99% credibility level using a window size and pace of 1.2 and 0.5, respectively, in the S axis [see Additional file 1]. We considered a gene as differentially expressed if all its replicates were above (up-regulated) or below (down-regulated) the dynamic cutoff level defined by the self-self experiments.

### Gene Enrichment and Functional Annotation Analysis

Given the lists of differentially expressed genes, we performed an ontology term enrichment analysis [38] using the DAVID 2.1 tool to find gene functional classes specifically associated with those lists [39]. In such analysis, the statistical association between being differentially expressed and belonging to a given category is accessed by the Fisher Exact test. The ontologies used were those defined by the KEGG database of metabolic pathways [40] and by the Gene Ontology Consortium [41]. The probe-to-GO and the probe-to-KEGG mapping were established based on the official annotation provided by the manufacturer. The analysis of gene expression levels in cancer and corresponding normal tissue was performed *in silico *using the SAGE Anatomic Viewer tool of the SAGE genie database [42].

### Real-time PCR quantification

Peripheral blood samples from 10 CML patients and 5 healthy volunteers were obtained after written informed consent approved by the Albert Einstein Hospital Internal Review Board. Mononuclear cells were purified for total RNA extraction as described above. One microgram of total RNA was used to synthesize cDNA by extension with oligo-dT primers and 200U of Superscript II Reverse Transcriptase (Invitrogen Life Technologies). Quantitative reverse-transcription polymerase chain reaction was performed by the Sybr-Green approach. Normalization of quantitative data was based on the expression of the housekeeping gene glyceraldehyde-3-phosphate dehydrogenase (GAPDH). Amplification specificity was assessed by dissociation curve analysis. Quantification was based on standard curves established with serial sample dilutions. Primer sequences and amplification conditions are provided [see Additional file 2].

### Statistical analysis

Median, mean, and standard deviation (SD) were calculated with Excel (Microsoft, Seattle, WA). Statistically significant differences among treatments were determined by the Student *t *test. All conclusions are based on at least 1% level of significance (*P *< 0.01) [43].

## Competing interests

The author(s) declare that they have no competing interests.

## Authors' contributions

OKO conceived the idea, designed and performed experiments, analyzed data and wrote the manuscript. ACSRC and LCM performed experiments. RZV analyzed microarray data and helped writing the manuscript. CAMF revised the manuscript. All authors read and approved the final manuscript.

## Supplementary Material

Additional file 1 supplemental figures. Series of four supplemental figures and respective legends relative to validation of the HTself approach for finding differentially expressed genes, flow cytometry analysis of CD34+/CD133+ cells, and functional classification of genes regulated in CD133+/CD34+ cells expanded *in vitro *in basal growth medium. Click here for file

Additional file 2 supplemental tables. Series of three supplemental tables containing the sequences of primers and amplification conditions for real-time PCR, as well as the enriched functional categories and corresponding genes up- and down-regulated in CD133+/CD34+ cells expanded *in vitro *under basal growth. Click here for file
