# Supplementary material for: Common molecular pathways involved in human CD133+/CD34+ progenitor cell expansion and cancer
Source: Cancer Cell Int. 2007 Jun 8;7:11. doi: 10.1186/1475-2867-7-11 (PMC1904434; doi:10.1186/1475-2867-7-11)

**LEGENDS TO SUPPLEMENTAL FIGURES**

[**Figure S1**](http://www.vision.ime.usp.br/~rvencio/UBC1/controles_estrad_vs_culture.png) **- Validation of the HTself approach for finding differentially expressed genes.** The MS-plot shows results from the 1h-estradiol comparison experiment [M = log2(1h estradiol/ 1h culture) and S = log2((1h estradiol + 1h culture)/2)]. The green cutoff curves were determined based on 3 self-self experiments usign the [HTself](http://blasto.iq.usp.br/~rvencio/HTself/) method. The highlighted points are the GE CodeLink spiked controls that should not present differential expression. As expected, the great majority of controls are inside the intensity-dependent cutoff curves.

[**Figure S2**](http://www.vision.ime.usp.br/~rvencio/UBC1/citometria.png) **- Flow cytometry analysis of CD34 and CD133 expression in mononucleated cells from human umbilical cord blood.**

[**Figure S3**](http://www.vision.ime.usp.br/~rvencio/UBC1/bars_GO_up.png) **- General functional classification of genes up-regulated in CD133+/CD34+ cells expanded *in vitro* in basal growth medium.** All classes, except marked with *, showed *p*-value < 0.05. Those marked with * showed *p*-value < 0.1. The *p*-values were obtained from the [DAVID 2.1](http://david.abcc.ncifcrf.gov/) method of statistical function classification tool.

[**Figure S4**](http://www.vision.ime.usp.br/~rvencio/UBC1/bars_GO_down.png) **- General functional classification of genes down-regulated in CD133+/CD34+ cells expanded *in vitro* in basal growth medium.** All classes, except marked with *, showed *p*-value < 0.05. Those marked with * showed *p*-value < 0.1. The *p*-values were obtained from the [DAVID 2.1](http://david.abcc.ncifcrf.gov/) method of statistical function classification tool.

**Figure S1**


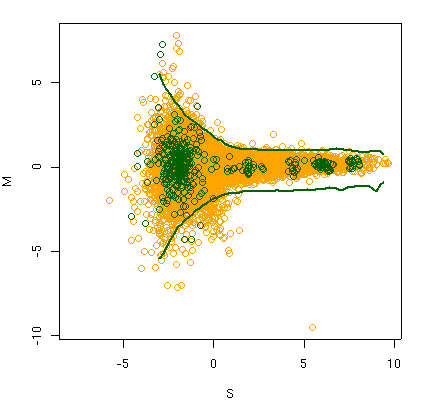


**Figure S2**


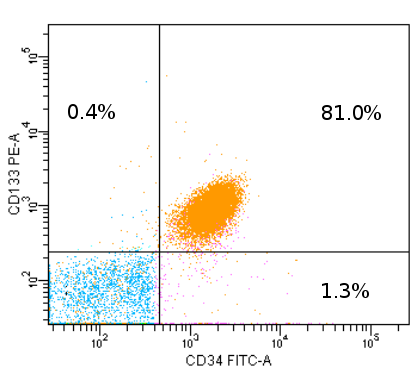

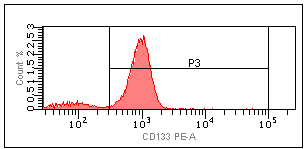


CD133+ cells

Isotypic control


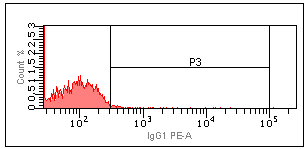


**Figure S3**


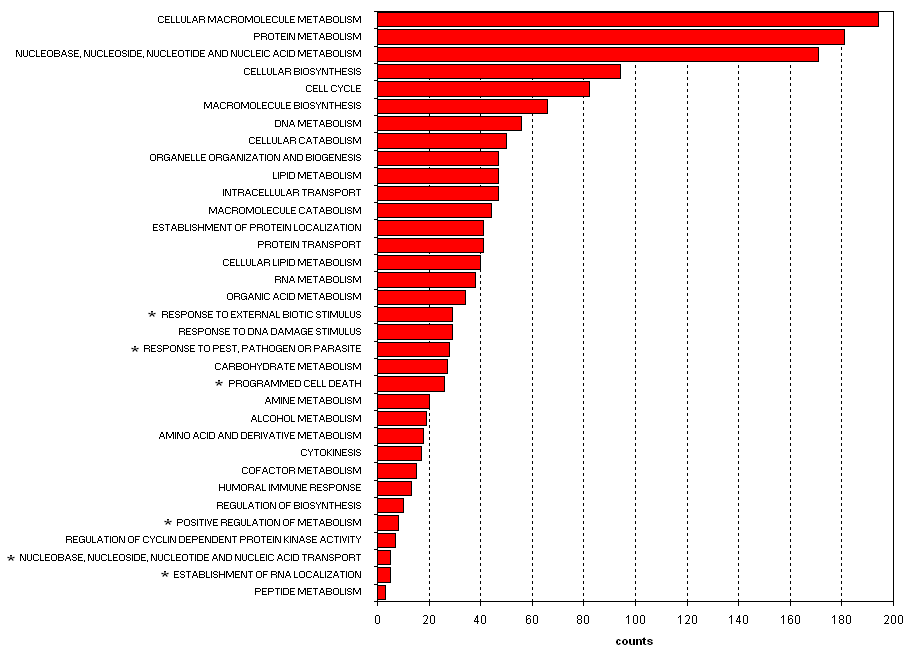


**Figure S4**


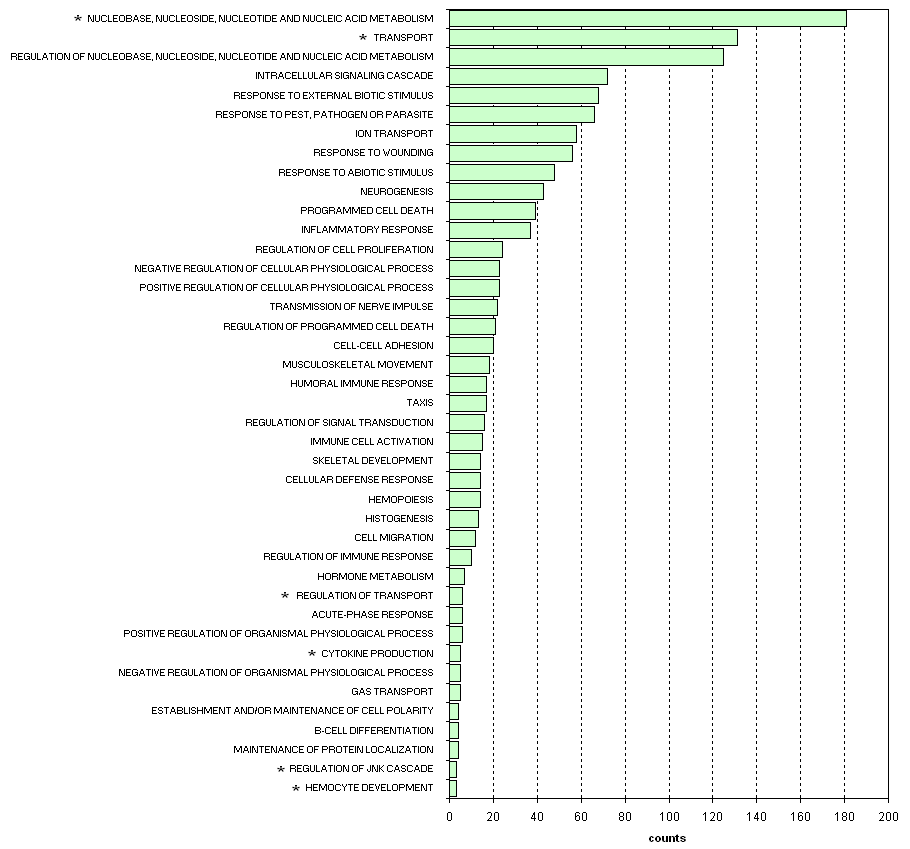

Supplement: Additional file 1 — supplemental figures. Series of four supplemental figures and respective legends relative to validation of the HTself approach for finding differentially expressed genes, flow cytometry analysis of CD34+/CD133+ cells, and functional classification of genes regulated in CD133+/CD34+ cells expanded in vitro in basal growth medium. [file 1475-2867-7-11-S1.doc]
