# Supplementary material for: Common molecular pathways involved in human CD133+/CD34+ progenitor cell expansion and cancer
Source: Cancer Cell Int. 2007 Jun 8;7:11. doi: 10.1186/1475-2867-7-11 (PMC1904434; doi:10.1186/1475-2867-7-11)
Supplement: Additional file 2 — supplemental tables. Series of three supplemental tables containing the sequences of primers and amplification conditions for real-time PCR, as well as the enriched functional categories and corresponding genes up- and down-regulated in CD133+/CD34+ cells expanded in vitro under basal growth. [file 1475-2867-7-11-S2.doc]

| **Supplemental table 1. Sequences of primers and amplification conditions for real-time PCR quantification:** | | | | |
| --- | --- | --- | --- | --- |
|  |  |  |  |  |
| **Gene** | **Forward sequence (5' →3')** | **Reverse sequence (5' →3')** | **Annealing Tº** | **Product length** |
| *MS4A6A* | CTC TAT CAA TCG CCA CAG | GTG AAT CAT GAT AAA AGT AAG | 54 | 210 |
| *FNBP3* | GCT CTC GAT CGG GGT CAG | CTC CTC TTC TTA CTT TTC TTC | 58 | 160 |
| *DOCK4* | GGA GAA CCC ACC CAG ACA | GTG AAC CAA AGA ATC AAA CAC | 56 | 164 |
| *Sparc-like 1* | GGC TCC TGG TGT TAG TTC | GAT CCT GCT CTT GGT TTC C | 56 | 164 |
| *Gli2  2* | CTT CCA CAG CAC CCA CAA C | CCC CGC GCC GTC CAC TC | 60 | 200 |
| *IRAK2* | CAGAGAATGGGGAAGGAAGG | GTCATCAGGGGCCAAAGAGC | 62 | 214 |
| *BCL10* | CAGATGGAGCCACGAACAAC | GATCTGGTGGCAAAGGAGG | 60 | 244 |
| *GAPDH* | GGAGAAGGCTGGGGCTCAT | GTCCTTCCACGATACCAAAGTT | 60 | 206 |

| **Supplemental table 2. Enriched functional categories and corresponding genes up-regulated in CD133+/CD34+ cells expanded in vitro in basal growth medium.** | | | | |
| --- | --- | --- | --- | --- |
| **PROTEIN BIOSYNTHESIS** | **Count: 41** | **P Value = 1.86E-40** | |  |
| Assession No. | Description | |  |  |
| Hs.420696 | mitochondrial ribosomal protein L39 | | | |
| Hs.5836 | mitochondrial ribosomal protein S23 | | | |
| Hs.431307 | mitochondrial ribosomal protein L40 | | | |
| Hs.534261 | mitochondrial ribosomal protein L46 | | | |
| Hs.55847 | mitochondrial ribosomal protein L51 | | | |
| Hs.265174 | ribosomal protein L32 | | |  |
| Hs.128201 | mitochondrial ribosomal protein S25 | | | |
| Hs.512676 | ribosomal protein S25 | | |  |
| Hs.44298 | mitochondrial ribosomal protein S17 | | | |
| Hs.346736 | mitochondrial ribosomal protein L30 | | | |
| Hs.521124 | mitochondrial ribosomal protein S28 | | | |
| Hs.352839 | mitochondrial ribosomal protein S15 | | | |
| Hs.109059 | mitochondrial ribosomal protein L12 | | | |
| Hs.182898 | J-type co-chaperone HSC20 | | | |
| Hs.325631 | AD023 protein | |  |  |
| Hs.301613 | JTV1 | |  |  |
| Hs.205163 | mitochondrial ribosomal protein L3 | | | |
| Hs.88977 | eukaryotic translation elongation factor 1 epsilon 1 | | | |
| Hs.532019 | mitochondrial ribosomal protein L1 | | | |
| Hs.369901 | mitochondrial ribosomal protein S36 | | | |
| Hs.247324 | mitochondrial ribosomal protein S14 | | | |
| Hs.355983 | basic leucine zipper and W2 domains 1 | | | |
| Hs.71787 | mitochondrial ribosomal protein S7 | | | |
| Hs.130849 | peptide deformylase-like protein | | | |
| Hs.515242 | mitochondrial ribosomal protein L34 | | | |
| Hs.462913 | mitochondrial ribosomal protein L45 | | | |
| Hs.356371 | ribosomal protein L28 | | |  |
| Hs.119316 | PET112-like (yeast) | | |  |
| Hs.279652 | mitochondrial ribosomal protein L4 | | | |
| Hs.438429 | ribosomal protein S19 | | |  |
| Hs.137367 | ribosomal protein S26 | | |  |
| Hs.78592 | eukaryotic translation initiation factor 2B, subunit 1 alpha, 26kDa | | | |
| Hs.44024 | mitochondrial ribosomal protein L19 | | | |
| Hs.378532 | HBS1-like (S. cerevisiae) | | |  |
| Hs.397609 | ribosomal protein S16 | | |  |
| Hs.523456 | mitochondrial ribosomal protein L17 | | | |
| Hs.420696 | mitochondrial ribosomal protein L39 | | | |
| Hs.528668 | ribosomal protein L6 | | |  |
| Hs.431307 | mitochondrial ribosomal protein L40 | | | |
| Hs.111286 | mitochondrial ribosomal protein S11 | | | |
| Hs.515879 | mitochondrial ribosomal protein L33 | | | |
|  |  |  |  |  |
| **TRANSCRIPTION** | **Count: 40** | **P Value = 8.75E-29** | | |
| Assession No. | Description | |  |  |
| Hs.19114 | high-mobility group box 3 | | |  |
| Hs.301756 | mediator of RNA polymerase II transcription, subunit 8 homolog (yeast) | | | |
| Hs.334518 | zinc finger protein 607 | | |  |
| Hs.183390 | zinc finger protein 613 | | |  |
| Hs.134859 | v-maf musculoaponeurotic fibrosarcoma oncogene homolog (avian) | | | |
| Hs.412951 | hypothetical protein FLJ10891 | | | |
| Hs.367725 | GATA binding protein 2 | | |  |
| Hs.89657 | TAF10 RNA polymerase II, TATA box binding protein (TBP)-associated factor, 30kDa | | | |
| Hs.409876 | zinc finger and BTB domain containing 24 | | | |
| Hs.466148 | nuclear receptor subfamily 2, group F, member 6 | | | |
| Hs.530479 | polyamine-modulated factor 1 | | | |
| Hs.436792 | LIM domain only 4 | | |  |
| Hs.479396 | recombining binding protein suppressor of hairless (Drosophila) | | | |
| Hs.170498 | zinc finger protein 609 | | |  |
| Hs.512934 | general transcription factor IIA, 2, 12kDa | | | |
| Hs.534483 | hypothetical protein MGC2941 | | | |
| Hs.500340 | thyroid hormone receptor interactor 4 | | | |
| Hs.521151 | hypothetical protein FLJ22301 | | | |
| Hs.492031 | hypothetical protein FLJ11011 | | | |
| Hs.210862 | T-box, brain, 1 | |  |  |
| Hs.351475 | polymerase (RNA) II (DNA directed) polypeptide K, 7.0kDa | | | |
| Hs.416358 | sal-like 2 (Drosophila) | | |  |
| Hs.107149 | chromosome 1 open reading frame 25 | | | |
| Hs.445272 | general transcription factor IIE, polypeptide 1, alpha 56kDa | | | |
| Hs.512740 | hypothetical protein FLJ14129 | | | |
| Hs.533357 | homeo box A3 | |  |  |
| Hs.166891 | regulatory factor X, 5 (influences HLA class II expression) | | | |
| Hs.248941 | TAF9 RNA polymerase II, TATA box binding protein (TBP)-associated factor, 32kDa | | | |
| Hs.437186 | polymerase (RNA) III (DNA directed) polypeptide K, 12.3 kDa | | | |
| Hs.520636 | RB-associated KRAB repressor | | | |
| Hs.374485 | HSPC038 protein | | |  |
| Hs.440332 | Ets2 repressor factor | | |  |
| Hs.500007 | activating signal cointegrator 1 complex subunit 1 | | | |
| Hs.493218 | zinc finger protein 7 (KOX 4, clone HF.16) | | | |
| Hs.546299 | small nuclear RNA activating complex, polypeptide 3, 50kDa | | | |
| Hs.524899 | sin3-associated polypeptide, 18kDa | | | |
| Hs.229641 | activated RNA polymerase II transcription cofactor 4 | | | |
| Hs.180402 | hypothetical protein FLJ23506 | | | |
| Hs.84928 | nuclear transcription factor Y, beta | | | |
| Hs.431043 | PBX/knotted 1 homeobox 1 | | | |
| **proteolysis and peptidolysis** | **Count: 21** | **P Value = 2.77E-28** | | |
| assession no. | Description | |  |  |
| Hs.251531 | proteasome (prosome, macropain) subunit, alpha type, 4 | | | |
| Hs.443837 | aminopeptidase puromycin sensitive | | | |
| Hs.467524 | ubiquitin specific protease 48 | | | |
| Hs.646 | carboxypeptidase A3 (mast cell) | | | |
| Hs.405479 | tryptase alpha/beta 1 | | |  |
| Hs.502914 | dipeptidylpeptidase 3 | | |  |
| Hs.252549 | cathepsin Z | |  |  |
| Hs.82793 | proteasome (prosome, macropain) subunit, beta type, 3 | | | |
| Hs.485246 | proteasome (prosome, macropain) subunit, alpha type, 5 | | | |
| Hs.517076 | protective protein for beta-galactosidase (galactosialidosis) | | | |
| Hs.928 | proteinase 3 (serine proteinase, neutrophil, Wegener granulomatosis autoantigen) | | | |
| Hs.535326 | asparaginase like 1 | | |  |
| Hs.9534 | SEC11-like 1 (S. cerevisiae) | | | |
| Hs.405479 | tryptase beta 2 | |  |  |
| Hs.148641 | cathepsin H | |  |  |
| Hs.381081 | proteasome (prosome, macropain) subunit, beta type, 9 | | | |
| Hs.11125 | signal peptidase complex subunit 1 homolog (S. cerevisiae) | | | |
| Hs.102798 | proteasome (prosome, macropain) subunit, alpha type, 1 | | | |
| Hs.132642 | zinc metallopeptidase (STE24 homolog, yeast) | | | |
| Hs.269254 | ubiquitin specific protease 49 | | | |
| Hs.458959 | F-box protein 22 | | |  |
|  |  |  |  |  |
| **KINASE ACTIVITY** | **Count: 19** | **P Value = 2.24E-23** | | |
| Assession No. | Description | |  |  |
| Hs.334562 | cell division cycle 2, G1 to S and G2 to M | | | |
| Hs.153704 | NIMA (never in mitosis gene a)-related kinase 2 | | | |
| Hs.234521 | mitogen-activated protein kinase-activated protein kinase 3 | | | |
| Hs.513520 | branched chain alpha-ketoacid dehydrogenase kinase | | | |
| Hs.182898 | CHK2 checkpoint homolog (S. pombe) | | | |
| Hs.30352 | ribosomal protein S6 kinase, 52kDa, polypeptide 1 | | | |
| Hs.466987 | protein kinase D2 | | |  |
| Hs.430742 | PFTAIRE protein kinase 1 | | |  |
| Hs.422662 | vaccinia related kinase 1 | | |  |
| Hs.382306 | cyclin-dependent kinase 8 | | |  |
| Hs.5158 | integrin-linked kinase | | |  |
| Hs.5158 | integrin-linked kinase-2 | | |  |
| Hs.434875 | calcium/calmodulin-dependent protein kinase I | | | |
| Hs.166071 | cyclin-dependent kinase 5 | | |  |
| Hs.371594 | MAP kinase interacting serine/threonine kinase 1 | | | |
| Hs.95577 | cyclin-dependent kinase 4 | | |  |
| Hs.24529 | CHK1 checkpoint homolog (S. pombe) | | | |
| Hs.119878 | hypothetical protein FLJ34389 | | | |
| Hs.502872 | mitogen-activated protein kinase kinase kinase 11 | | | |
|  |  |  |  |  |
| **RNA PROCESSING** | **Count: 15** | **P Value = 2.78E-19** | | |
| Assession No. | Description | |  |  |
| Hs.204475 | HIV TAT specific factor 1 | | |  |
| Hs.249996 | pleiotropic regulator 1 (PRL1homolog, Arabidopsis) | | | |
| Hs.533862 | survival of motor neuron protein interacting protein 1 | | | |
| Hs.508848 | heterogeneous nuclear ribonucleoprotein C (C1/C2) | | | |
| Hs.143818 | gem (nuclear organelle) associated protein 6 | | | |
| Hs.5086 | hypothetical protein MGC10433 | | | |
| Hs.487774 | heterogeneous nuclear ribonucleoprotein A2/B1 | | | |
| Hs.369606 | cleavage and polyadenylation specific factor 6, 68kDa | | | |
| Hs.464734 | small nuclear ribonucleoprotein D1 polypeptide 16kDa | | | |
| Hs.465498 | thioredoxin-like 4A | | |  |
| Hs.79110 | nucleolin | |  |  |
| Hs.356549 | small nuclear ribonucleoprotein D3 polypeptide 18kDa | | | |
| Hs.105465 | small nuclear ribonucleoprotein polypeptide F | | | |
| Hs.367842 | MKI67 (FHA domain) interacting nucleolar phosphoprotein | | | |
| Hs.309763 | G-rich RNA sequence binding factor 1 | | | |
|  |  |  |  |  |
| **OXIDATIVE PHOSPHORYLATION** | **Count: 14** | **P Value = 7.41E-18** | | |
| Assession No. | Description | |  |  |
| Hs.189716 | NADH dehydrogenase (ubiquinone) 1, alpha/beta subcomplex, 1, 8kDa | | | |
| Hs.131255 | ubiquinol-cytochrome c reductase binding protein | | | |
| Hs.106529 | NADH dehydrogenase (ubiquinone) 1 alpha subcomplex, assembly factor 1 | | | |
| Hs.324250 | NADH dehydrogenase (ubiquinone) 1 beta subcomplex, 2, 8kDa | | | |
| Hs.284292 | ubiquinol-cytochrome c reductase complex 7.2kDa protein | | | |
| Hs.50098 | NADH dehydrogenase (ubiquinone) 1 alpha subcomplex, 4, 9kDa | | | |
| Hs.518994 | NADH dehydrogenase (ubiquinone) 1, subcomplex unknown, 1, 6kDa | | | |
| Hs.495039 | NADH dehydrogenase (ubiquinone) 1 alpha subcomplex, 8, 19kDa | | | |
| Hs.471207 | NADH dehydrogenase (ubiquinone) Fe-S protein 1, 75kDa | | | |
| Hs.473937 | NADH dehydrogenase (ubiquinone) flavoprotein 3, 10kDa | | | |
| Hs.90443 | NADH dehydrogenase (ubiquinone) Fe-S protein 8, 23kDa | | | |
| Hs.183435 | NADH dehydrogenase (ubiquinone) 1 beta subcomplex, 1, 7kDa | | | |
| Hs.464572 | NADH dehydrogenase (ubiquinone) flavoprotein 2, 24kDa | | | |
| Hs.528803 | ubiquinol-cytochrome c reductase core protein II | | | |
|  |  |  |  |  |
| **RNA MODIFICATION** | **Count: 9** | **P Value = 1.06E-19** | | |
| Assession No. | Description | |  |  |
| Hs.505231 | CGI-04 protein | |  |  |
| Hs.503389 | hypothetical protein FLJ23441 | | | |
| Hs.23111 | phenylalanine-tRNA synthetase-like, alpha subunit | | | |
| Hs.508292 | hypothetical protein FLJ10514 | | | |
| Hs.531176 | seryl-tRNA synthetase | | |  |
| Hs.497788 | glutamyl-prolyl-tRNA synthetase | | | |
| Hs.445403 | isoleucine-tRNA synthetase | | | |
| Hs.432560 | histidyl-tRNA synthetase-like | | | |
| Hs.526975 | leucyl-tRNA synthetase 2, mitochondrial | | | |
|  |  |  |  |  |
| **INTRACELLULAR PROTEIN TRANSPORT** | **Count: 7** | **P Value = 3.74E-9** | |  |
| Assession No. | Description | |  |  |
| Hs.528653 | chromosome 14 open reading frame 108 | | | |
| Hs.491351 | clathrin, heavy polypeptide (Hc) | | | |
| Hs.518460 | adaptor-related protein complex 2, mu 1 subunit | | | |
| Hs.71040 | adaptor-related protein complex 1, mu 1 subunit | | | |
| Hs.522114 | clathrin, light polypeptide (Lca) | | | |
| Hs.500104 | adaptor-related protein complex 3, mu 1 subunit | | | |
| Hs.33642 | archain 1 | |  |  |
|  |  |  |  |  |
| **WD REPEAT** | **Count: 7** | **P Value = 1.54E-10** | |  |
| Assession No. | Description | |  |  |
| Hs.461113 | cirrhosis, autosomal recessive 1A (cirhin) | | | |
| Hs.249996 | pleiotropic regulator 1 (PRL1homolog, Arabidopsis) | | | |
| Hs.204773 | methylosome protein 50 | | |  |
| Hs.73291 | WD repeat domain 12 | | |  |
| Hs.418533 | BUB3 budding uninhibited by benzimidazoles 3 homolog (yeast) | | | |
| Hs.201375 | WD repeat domain 3 | | |  |
| Hs.495755 | retinoblastoma binding protein 7 | | | |
|  |  |  |  |  |
| **DNA METABOLISM** | **Count: 6** | **P Value = 2.52E-6** | |  |
| Assession No. | Description | |  |  |
| Hs.284137 | chromosome 9 open reading frame 76 | | | |
| Hs.240170 | hypothetical protein MGC2731 | | | |
| Hs.134491 | oligonucleotide/oligosaccharide-binding fold containing 1 | | | |
| Hs.511754 | S-phase response (cyclin-related) | | | |
| Hs.368022 | hypothetical protein FLJ11200 | | | |
| Hs.24088 | hypothetical protein FLJ20125 | | | |
|  |  |  |  |  |
| **SMALL GTPASE MEDIATED SIGNAL TRANSDUCTION** | **Count: 6** | **P Value = 1.24E-9** | |  |
| Assession No. | Description | |  |  |
| Hs.151536 | RAB13, member RAS oncogene family | | | |
| Hs.495704 | RAB9A, member RAS oncogene family | | | |
| Hs.25362 | ADP-ribosylation factor-like 8 | | | |
| Hs.5947 | RAB8A, member RAS oncogene family | | | |
| Hs.321541 | RAB11A, member RAS oncogene family | | | |
| Hs.250009 | ADP-ribosylation factor-like 10C | | | |
|  |  |  |  |  |
| **ATP METABOLISM** | **Count: 6** | **P Value = 2.11E-11** | | |
| Assession No. | Description | |  |  |
| Hs.372429 | ATPase, H+ transporting, lysosomal 42kDa, V1 subunit C isoform 2 | | | |
| Hs.85539 | ATP synthase, H+ transporting, mitochondrial F0 complex, subunit e | | | |
| Hs.514870 | ATP synthase, H+ transporting, mitochondrial F0 complex, subunit b, isoform 1 | | | |
| Hs.484188 | ATPase, H+ transporting, lysosomal 9kDa, V0 subunit e | | | |
| Hs.282925 | ATP synthase, H+ transporting, mitochondrial F0 complex, subunit f, isoform 2 | | | |
| Hs.85539 | ATP synthase, H+ transporting, mitochondrial F0 complex, subunit e | | | |
|  |  |  |  |  |
| **PROTEIN UBIQUITINATION** | **Count: 6** | **P Value = 3.5E-9** | |  |
| Assession No. | Description | |  |  |
| Hs.124186 | ring finger protein 2 | | |  |
| Hs.279474 | makorin, ring finger protein, 2 | | | |
| Hs.22146 | ring finger protein 144 | | |  |
| Hs.306769 | RUN and FYVE domain containing 1 | | | |
| Hs.485041 | tripartite motif-containing 26 | | | |
| Hs.125300 | tripartite motif-containing 6 | | | |
|  |  |  |  |  |
| **TRANSLATION, RIBOSOMAL STRUCTURE AND BIOGENESIS** | **Count: 5** | **P Value = 6.3E-3** | |  |
| Assession No. | Description | |  |  |
| Hs.420696 | mitochondrial ribosomal protein L39 | | | |
| Hs.5836 | mitochondrial ribosomal protein S23 | | | |
| Hs.431307 | mitochondrial ribosomal protein L40 | | | |
| Hs.534261 | mitochondrial ribosomal protein L46 | | | |
| Hs.55847 | mitochondrial ribosomal protein L51 | | | |
|  |  |  |  |  |
| **PROTEIN FOLDING** | **Count: 5** | **P Value = 4.58E-8** | |  |
| Assession No. | Description | |  |  |
| Hs.1197 | heat shock 10kDa protein 1 (chaperonin 10) | | | |
| Hs.368149 | chaperonin containing TCP1, subunit 7 (eta) | | | |
| Hs.189772 | chaperonin containing TCP1, subunit 2 (beta) | | | |
| Hs.446374 | HLA class II region expressed gene KE2 | | | |
| Hs.491494 | chaperonin containing TCP1, subunit 3 (gamma) | | | |

| **Supplemental table 3. Enriched functional categories and corresponding genes down-regulated in CD133+/CD34+ cells expanded in vitro in basal growth medium.** | | | |
| --- | --- | --- | --- |
|  | | | |
| **TRANSCRIPTION** | | **Count: 111** | **P Value = 0E0** |
| Assession No. | Description |  |  |
| Hs.282079 | Mix1 homeobox-like 1 (Xenopus laevis) | | |
| Hs.254097 | hypothetical protein BC012187 | | |
| Hs.310575 | homeo box C5 | |  |
| Hs.517296 | v-ets erythroblastosis virus E26 oncogene homolog 2 (avian) | | |
| Hs.1004 | bromodomain and PHD finger containing, 1 | | |
| Hs.270869 | zinc finger protein 410 | |  |
| Hs.166204 | PHD finger protein 1 | |  |
| Hs.510396 | B-cell CLL/lymphoma 11B (zinc finger protein) | | |
| Hs.288042 | hypothetical protein FLJ14299 | | |
| Hs.434142 | synovial sarcoma, X breakpoint 1 | | |
| Hs.20084 | retinoid X receptor, alpha | |  |
| Hs.376984 | SRY (sex determining region Y)-box 10 | | |
| Hs.514292 | homeo box B8 | |  |
| Hs.499453 | zinc finger protein 11b (KOX 2) | | |
| Hs.444409 | MADS box transcription enhancer factor 2, polypeptide C (myocyte enhancer factor 2C) | | |
| Hs.77637 | homeo box A4 | |  |
| Hs.107740 | Kruppel-like factor 2 (lung) | | |
| Hs.157429 | SRY (sex determining region Y)-box 3 | | |
| Hs.307924 | male-specific lethal 3-like 1 (Drosophila) | | |
| Hs.283416 | paired related homeobox 1 | | |
| Hs.196927 | iroquois homeobox protein 4 | | |
| Hs.165258 | nuclear receptor subfamily 4, group A, member 2 | | |
| Hs.511316 | GA binding protein transcription factor, beta subunit 2, 47kDa | | |
| Hs.435761 | protein inhibitor of activated STAT, 3 | | |
| Hs.73677 | regulatory factor X, 1 (influences HLA class II expression) | | |
| Hs.467210 | zinc finger protein 83 (HPF1) | | |
| Hs.435535 | zinc finger protein 395 | |  |
| Hs.91531 | myeloid/lymphoid or mixed-lineage leukemia (trithorax homolo ... | | |
| Hs.37128 | transcription factor similar to D. melanogaster homeodomain protein lady bird late | | |
| Hs.373550 | TGFB-induced factor (TALE family homeobox) | | |
| Hs.463375 | zinc finger protein 652 | |  |
| Hs.170019 | runt-related transcription factor 3 | | |
| Hs.471991 | metal-regulatory transcription factor 1 | | |
| Hs.367725 | GATA binding protein 2 | |  |
| Hs.369519 | TAF4b RNA polymerase II, TATA box binding protein (TBP)-associated factor, 105kDa | | |
| Hs.466257 | pre-B-cell leukemia transcription factor 4 | | |
| Hs.48029 | snail homolog 1 (Drosophila) | | |
| Hs.124503 | transcription factor 8 (represses interleukin 2 expression) | | |
| Hs.250666 | hairy and enhancer of split 1, (Drosophila) | | |
| Hs.509545 | pre-B-cell leukemia transcription factor 2 | | |
| Hs.154029 | hairy and enhancer of split 4 (Drosophila) | | |
| Hs.517617 | v-maf musculoaponeurotic fibrosarcoma oncogene homolog F (avian) | | |
| Hs.517418 | zinc finger protein 74 (Cos52) | | |
| Hs.94367 | thyroid transcription factor 1 | | |
| Hs.156471 | transcription factor CP2-like 1 | | |
| Hs.515114 | hypothetical protein MGC15716 | | |
| Hs.513100 | zinc finger and SCAN domain containing 2 | | |
| Hs.205392 | zinc finger protein 507 | |  |
| Hs.491805 | thymus high mobility group box protein TOX | | |
| Hs.28346 | glial cells missing homolog 1 (Drosophila) | | |
| Hs.67928 | E74-like factor 3 (ets domain transcription factor, epithelial-specific ) | | |
| Hs.1497 | retinoic acid receptor, gamma | | |
| Hs.177688 | zinc finger and SCAN domain containing 5 | | |
| Hs.37034 | homeo box A5 | |  |
| Hs.59757 | zinc finger protein 281 | |  |
| Hs.112968 | forkhead box E3 | |  |
| Hs.222802 | chromosome 21 open reading frame 7 | | |
| Hs.185674 | zinc finger protein 331 | |  |
| Hs.504609 | inhibitor of DNA binding 1, dominant negative helix-loop-helix protein | | |
| Hs.820 | homeo box C6 | |  |
| Hs.298658 | Kruppel-like factor 3 (basic) | | |
| Hs.11713 | E74-like factor 5 (ets domain transcription factor) | | |
| Hs.469633 | LIM and senescent cell antigen-like domains 3 | | |
| Hs.435001 | Kruppel-like factor 10 | |  |
| Hs.241523 | zinc finger protein 312 | |  |
| Hs.234759 | FEV (ETS oncogene family) | | |
| Hs.357901 | SRY (sex determining region Y)-box 4 | | |
| Hs.515872 | zinc finger protein 513 | |  |
| Hs.211588 | POU domain, class 4, transcription factor 2 | | |
| Hs.307905 | v-rel reticuloendotheliosis viral oncogene homolog B | | |
| Hs.99430 | testis zinc finger protein | |  |
| Hs.310893 | RNA-binding protein pippin | | |
| Hs.445340 | orthodenticle homolog 1 (Drosophila) | | |
| Hs.724 | thyroid hormone receptor, alpha (erythroblastic leukemia vir ... | | |
| Hs.517106 | CCAAT/enhancer binding protein (C/EBP), beta | | |
| Hs.386324 | zinc finger protein 394 | |  |
| Hs.517557 | zinc finger protein 278 | |  |
| Hs.247744 | cAMP responsive element binding protein 3-like 3 | | |
| Hs.502330 | GLI-Kruppel family member HKR3 | | |
| Hs.276916 | nuclear receptor subfamily 1, group D, member 1 | | |
| Hs.460 | activating transcription factor 3 | | |
| Hs.32938 | insulin promoter factor 1, homeodomain transcription factor | | |
| Hs.20131 | nuclear receptor subfamily 6, group A, member 1 | | |
| Hs.303808 | chromosome 2 open reading frame 3 | | |
| Hs.92282 | paired-like homeodomain transcription factor 2 | | |
| Hs.227098 | glial cells missing homolog 2 (Drosophila) | | |
| Hs.72981 | neurogenic differentiation 1 | | |
| Hs.820 | homeo box C4 | |  |
| Hs.289292 | forkhead box L2 | |  |
| Hs.436061 | interferon regulatory factor 1 | | |
| Hs.446318 | homeo box A7 | |  |
| Hs.169487 | v-maf musculoaponeurotic fibrosarcoma oncogene homolog B (avian) | | |
| Hs.55481 | zinc finger protein 165 | |  |
| Hs.524920 | zinc finger protein 91 homolog (mouse) | | |
| Hs.497520 | ELK4, ETS-domain protein (SRF accessory protein 1) | | |
| Hs.510172 | human immunodeficiency virus type I enhancer binding protein 2 | | |
| Hs.249170 | ventral anterior homeobox 2 | | |
| Hs.520459 | general transcription factor II, i | | |
| Hs.504115 | tripartite motif-containing 29 | | |
| Hs.192221 | elongation factor, RNA polymerase II, 2 | | |
| Hs.319171 | nuclear factor of kappa light polypeptide gene enhancer in B-cells inhibitor, zeta | | |
| Hs.272409 | T-box 21 |  |  |
| Hs.507355 | zinc finger protein 10 (KOX 1) | | |
| Hs.205627 | rearranged L-myc fusion sequence | | |
| Hs.424414 | msh homeo box homolog 1 (Drosophila) | | |
| Hs.285313 | Kruppel-like factor 6 | |  |
| Hs.147765 | zinc finger protein 415 | |  |
| Hs.30385 | myeloid/lymphoid or mixed-lineage leukemia (trithorax homolo ... | | |
| Hs.171825 | basic helix-loop-helix domain containing, class B, 2 | | |
| Hs.89404 | msh homeo box homolog 2 (Drosophila) | | |
| Hs.458401 | hypothetical protein FLJ12363 | | |
|  |  |  |  |
| **SIGNAL TRANSDUCTION** | | **Count: 42** | **P Value = 6.18E-26** |
| Assession No. | Description |  |  |
| Hs.114545 | G protein-coupled receptor 55 | | |
| Hs.247861 | olfactory receptor, family 11, subfamily A, member 1 | | |
| Hs.368632 | adrenergic, alpha-1B-, receptor | | |
| Hs.158329 | G protein-coupled receptor 50 | | |
| Hs.118513 | MAS-related GPR, member F | | |
| Hs.73037 | cannabinoid receptor 2 (macrophage) | | |
| Hs.389103 | glucagon-like peptide 1 receptor | | |
| Hs.248056 | G protein-coupled receptor 43 | | |
| Hs.148685 | G protein-coupled receptor, family C, group 5, member B | | |
| Hs.118118 | transmembrane 4 superfamily member 9 | | |
| Hs.14468 | peter pan homolog (Drosophila) | | |
| Hs.22180 | 5-hydroxytryptamine (serotonin) receptor 6 | | |
| Hs.525534 | G protein-coupled receptor 68 | | |
| Hs.546396 | purinergic receptor P2Y, G-protein coupled, 13 | | |
| Hs.534399 | opsin 3 (encephalopsin, panopsin) | | |
| Hs.248160 | somatostatin receptor 1 | |  |
| Hs.416024 | chromosome 20 open reading frame 98 | | |
| Hs.99855 | formyl peptide receptor-like 1 | | |
| Hs.153381 | Duffy blood group | |  |
| Hs.185692 | chemokine binding protein 2 | | |
| Hs.287490 | G protein-coupled receptor 157 | | |
| Hs.258574 | olfactory receptor, family 2, subfamily C, member 1 | | |
| Hs.283922 | opsin 4 (melanopsin) | |  |
| Hs.271809 | G protein-coupled receptor 161 | | |
| Hs.73883 | gastrin-releasing peptide receptor | | |
| Hs.352218 | oxoglutarate (alpha-ketoglutarate) receptor 1 | | |
| Hs.512836 | G protein-coupled receptor 52 | | |
| Hs.248118 | G protein-coupled receptor 8 | | |
| Hs.512691 | G protein-coupled receptor 135 | | |
| Hs.91622 | neuronal pentraxin receptor | | |
| Hs.458425 | G protein-coupled receptor 109B | | |
| Hs.192720 | urotensin 2 receptor | |  |
| Hs.88269 | G protein-coupled receptor 18 | | |
| Hs.158351 | galanin receptor 2 | |  |
| Hs.102119 | opsin 1 (cone pigments), short-wave-sensitive (color blindness, tritan) | | |
| Hs.248202 | glucagon-like peptide 2 receptor | | |
| Hs.248124 | G protein-coupled receptor 31 | | |
| Hs.248145 | melanocortin 5 receptor | |  |
| Hs.272191 | galanin receptor 1 | |  |
| Hs.446438 | G protein-coupled receptor, family C, group 5, member C | | |
| Hs.446879 | olfactory receptor, family 4, subfamily D, member 2 | | |
| Hs.170146 | relaxin 3 receptor 1 | |  |
|  |  |  |  |
| **KINASE ACTIVITY** | | **Count: 33** | **P Value = 4.35E-41** |
| Assession No. | Description |  |  |
| Hs.444947 | tribbles homolog 1 (Drosophila) | | |
| Hs.463978 | mitogen-activated protein kinase kinase 6 | | |
| Hs.497512 | hypothetical protein DKFZp434J037 | | |
| Hs.442592 | casein kinase 1, alpha 1 | |  |
| Hs.203420 | tyrosine kinase, non-receptor, 1 | | |
| Hs.153640 | polo-like kinase 3 (Drosophila) | | |
| Hs.88297 | serine/threonine kinase 17b (apoptosis-inducing) | | |
| Hs.133062 | serine/threonine kinase 32B | | |
| Hs.291623 | TAO kinase 2 | |  |
| Hs.333907 | protein kinase C, eta | |  |
| Hs.73962 | EPH receptor A7 | |  |
| Hs.498570 | protein kinase C, theta | |  |
| Hs.513645 | p21(CDKN1A)-activated kinase 6 | | |
| Hs.369265 | interleukin-1 receptor-associated kinase 3 | | |
| Hs.488293 | epidermal growth factor receptor (erythroblastic leukemia vi ... | | |
| Hs.508514 | serine/threonine kinase 24 (STE20 homolog, yeast) | | |
| Hs.477070 | casein kinase 1, delta | |  |
| Hs.517493 | adrenergic, beta, receptor kinase 2 | | |
| Hs.124027 | selenophosphate synthetase 1 | | |
| Hs.509067 | platelet-derived growth factor receptor, beta polypeptide | | |
| Hs.424542 | interleukin-1 receptor-associated kinase 2 | | |
| Hs.514681 | mitogen-activated protein kinase kinase 4 | | |
| Hs.491322 | PTK2B protein tyrosine kinase 2 beta | | |
| Hs.390729 | v-erb-a erythroblastic leukemia viral oncogene homolog 4 (avian) | | |
| Hs.476052 | SNF-1 related kinase | |  |
| Hs.546277 | protein kinase, cGMP-dependent, type II | | |
| Hs.471768 | SINK-homologous serine/threonine kinase | | |
| Hs.103755 | receptor-interacting serine-threonine kinase 2 | | |
| Hs.20573 | insulin-like growth factor 1 receptor | | |
| Hs.352370 | serine/threonine kinase 32A | | |
| Hs.506415 | PCTAIRE protein kinase 2 | | |
| Hs.390788 | protein kinase, X-linked | |  |
| Hs.178695 | mitogen-activated protein kinase 13 | | |
|  |  |  |  |
| **PROTEOLYSIS AND PEPTIDOLYSIS** |  | **Count: 24** | **P Value = 1.93E-32** |
| Assession No. | Description |  |  |
| Hs.404089 | suppressor of fused homolog (Drosophila) | | |
| Hs.277937 | granzyme K (serine protease, granzyme 3; tryptase II) | | |
| Hs.375129 | matrix metalloproteinase 3 (stromelysin 1, progelatinase) | | |
| Hs.42400 | ubiquitin specific protease 12 | | |
| Hs.435765 | glutamyl aminopeptidase (aminopeptidase A) | | |
| Hs.500842 | meningioma expressed antigen 5 (hyaluronidase) | | |
| Hs.36989 | coagulation factor VII (serum prothrombin conversion accelerator) | | |
| Hs.458355 | complement component 1, s subcomponent | | |
| Hs.280658 | protease, serine, 33 | |  |
| Hs.302383 | matrix metalloproteinase 20 (enamelysin) | | |
| Hs.78977 | proprotein convertase subtilisin/kexin type 1 | | |
| Hs.297413 | matrix metalloproteinase 9 (gelatinase B, 92kDa gelatinase, type IV collagenase) | | |
| Hs.170499 | X-prolyl aminopeptidase (aminopeptidase P) 2, membrane-bound | | |
| Hs.534221 | a disintegrin-like and metalloprotease (reprolysin type) wit ... | | |
| Hs.79033 | glutaminyl-peptide cyclotransferase (glutaminyl cyclase) | | |
| Hs.144875 | hypothetical protein FLJ14442 | | |
| Hs.494321 | ATP/GTP binding protein 1 | | |
| Hs.195080 | endothelin converting enzyme 1 | | |
| Hs.1867 | progastricsin (pepsinogen C) | | |
| Hs.367767 | protease, serine, 2 (trypsin 2) | | |
| Hs.83169 | matrix metalloproteinase 1 (interstitial collagenase) | | |
| Hs.8709 | chymotrypsin C (caldecrin) | | |
| Hs.348264 | granzyme H (cathepsin G-like 2, protein h-CCPX) | | |
| Hs.46720 | transmembrane protease, serine 5 (spinesin) | | |
|  |  |  |  |
| **RESPONSE TO BIOTIC STIMULUS** |  | **Count: 22** | **P Value = 4.55E-11** |
| Assession No. | Description |  |  |
| Hs.268510 | killer cell lectin-like receptor subfamily K, member 1 | | |
| Hs.262150 | CD22 antigen | |  |
| Hs.488237 | CD160 antigen | |  |
| Hs.89575 | CD79B antigen (immunoglobulin-associated beta) | | |
| Hs.283022 | triggering receptor expressed on myeloid cells 1 | | |
| Hs.158315 | interleukin 18 receptor accessory protein | | |
| Hs.116481 | CD72 antigen | |  |
| Hs.436677 | NFAT activation molecule 1 | | |
| Hs.194721 | natural cytotoxicity triggering receptor 2 | | |
| Hs.74082 | killer cell lectin-like receptor subfamily C, member 2 | | |
| Hs.525534 | G protein-coupled receptor 68 | | |
| Hs.208854 | CD69 antigen (p60, early T-cell activation antigen) | | |
| Hs.50716 | signal-regulatory protein beta 2 | | |
| Hs.437229 | glycoprotein A33 (transmembrane) | | |
| Hs.546263 | killer cell immunoglobulin-like receptor, three domains, long cytoplasmic tail, 2 | | |
| Hs.484703 | CD83 antigen (activated B lymphocytes, immunoglobulin superfamily) | | |
| Hs.36972 | CD7 antigen (p41) | |  |
| Hs.488007 | acyloxyacyl hydrolase (neutrophil) | | |
| Hs.128846 | protein tyrosine phosphatase, non-receptor type substrate 1 | | |
| Hs.153381 | Duffy blood group | |  |
| Hs.79630 | CD79A antigen (immunoglobulin-associated alpha) | | |
| Hs.74082 | killer cell lectin-like receptor subfamily C, member 3 | | |
|  |  |  |  |
| **ION TRANSPORT** | | **Count: 17** | **P Value = 1E-5** |
| Assession No. | Description |  |  |
| Hs.512681 | sodium channel, nonvoltage-gated 1, delta | | |
| Hs.540696 | solute carrier family 6 (neurotransmitter transporter, creatine), member 8 | | |
| Hs.327179 | solute carrier family 17 (sodium phosphate), member 3 | | |
| Hs.130101 | solute carrier family 5 (low affinity glucose cotransporter), member 4 | | |
| Hs.103983 | solute carrier family 5 (sodium iodide symporter), member 5 | | |
| Hs.512681 | sodium channel, nonvoltage-gated 1, delta | | |
| Hs.225671 | hyperpolarization activated cyclic nucleotide-gated potassium channel 4 | | |
| Hs.248139 | potassium voltage-gated channel, shaker-related subfamily, member 2 | | |
| Hs.473058 | potassium voltage-gated channel, KQT-like subfamily, member 4 | | |
| Hs.102308 | potassium inwardly-rectifying channel, subfamily J, member 8 | | |
| Hs.458267 | potassium voltage-gated channel, shaker-related subfamily, member 4 | | |
| Hs.248101 | cholinergic receptor, nicotinic, gamma polypeptide | | |
| Hs.32973 | glycine receptor, beta | |  |
| Hs.156289 | cholinergic receptor, nicotinic, delta polypeptide | | |
| Hs.129783 | sodium channel, voltage-gated, type II, beta | | |
| Hs.519693 | glutamate receptor, ionotropic, AMPA 1 | | |
| Hs.367799 | glutamate receptor, ionotropic, kainate 5 | | |
|  |  |  |  |
| **CELL-CELL ADHESION** | | **Count: 15** | **P Value = 1.33E-27** |
| Assession No. | Description |  |  |
| Hs.368160 | protocadherin gamma subfamily A, 4 | | |
| Hs.368160 | protocadherin gamma subfamily A, 10 | | |
| Hs.368160 | protocadherin gamma subfamily A, 12 | | |
| Hs.368160 | protocadherin gamma subfamily C, 3 | | |
| Hs.368160 | protocadherin gamma subfamily A, 7 | | |
| Hs.368160 | protocadherin gamma subfamily A, 5 | | |
| Hs.89436 | cadherin 17, LI cadherin (liver-intestine) | | |
| Hs.191842 | cadherin 3, type 1, P-cadherin (placental) | | |
| Hs.232819 | protocadherin 15 | |  |
| Hs.368160 | protocadherin gamma subfamily A, 6 | | |
| Hs.148090 | cadherin 15, M-cadherin (myotubule) | | |
| Hs.368160 | protocadherin gamma subfamily A, 11 | | |
| Hs.368160 | protocadherin gamma subfamily A, 8 | | |
| Hs.368160 | protocadherin gamma subfamily B, 7 | | |
| Hs.546421 | cadherin-like 23 | |  |
|  |  |  |  |
| **CHEMOKINE RECEPTOR BINDING** |  | **Count: 11** | **P Value = 2.39E-26** |
| Assession No. | Description |  |  |
| Hs.75765 | chemokine (C-X-C motif) ligand 2 | | |
| Hs.514821 | chemokine (C-C motif) ligand 5 | | |
| Hs.10458 | chemokine (C-C motif) ligand 16 | | |
| Hs.512305 | chemokine (C-C motif) ligand 4-like | | |
| Hs.546295 | chemokine (C motif) ligand 1 | | |
| Hs.483444 | chemokine (C-X-C motif) ligand 14 | | |
| Hs.546295 | chemokine (C motif) ligand 2 | | |
| Hs.164021 | chemokine (C-X-C motif) ligand 6 (granulocyte chemotactic protein 2) | | |
| Hs.531668 | chemokine (C-X3-C motif) ligand 1 | | |
| Hs.89690 | chemokine (C-X-C motif) ligand 3 | | |
| Hs.251526 | chemokine (C-C motif) ligand 7 | | |
|  |  |  |  |
| **UBIQUITIN CYCLE** | | **Count: 9** | **P Value = 3.1E-12** |
| Assession No. | Description |  |  |
| Hs.435761 | protein inhibitor of activated STAT, 3 | | |
| Hs.456557 | hypothetical protein FLJ10597 | | |
| Hs.211374 | hypothetical protein MGC4734 | | |
| Hs.8375 | TNF receptor-associated factor 4 | | |
| Hs.188553 | retinoblastoma binding protein 6 | | |
| Hs.108106 | ubiquitin-like, containing PHD and RING finger domains, 1 | | |
| Hs.12256 | midline 2 |  |  |
| Hs.151237 | ring finger protein 122 | |  |
| Hs.484738 | myosin regulatory light chain interacting protein | | |
|  |  |  |  |
| **PROTEIN AMINO ACID GLYCOSYLATION** |  | **Count: 8** | **P Value = 1.28E-15** |
| Assession No. | Description |  |  |
| Hs.308628 | sialyltransferase 8D (alpha-2, 8-polysialyltransferase) | | |
| Hs.194710 | glucosaminyl (N-acetyl) transferase 3, mucin type | | |
| Hs.69009 | UDP-GlcNAc:betaGal beta-1,3-N-acetylglucosaminyltransferase 3 | | |
| Hs.115903 | mannosyl (alpha-1,6-)-glycoprotein beta-1,6-N-acetyl-glucosaminyltransferase | | |
| Hs.298923 | sialyltransferase 8C (alpha2,3Galbeta1,4GlcNAcalpha 2,8-sialyltransferase) | | |
| Hs.132989 | protein-O-mannosyltransferase 2 | | |
| Hs.108973 | dolichyl-phosphate mannosyltransferase polypeptide 2, regulatory subunit | | |
| Hs.207459 | sialyltransferase 1 (beta-galactoside alpha-2,6-sialyltransferase) | | |
|  |  |  |  |
| **INTEGRAL TO MEMBRANE** | | **Count: 7** | **P Value = 1.61E-3** |
| Assession No. | Description |  |  |
| Hs.134074 | solute carrier family 35, member E1 | | |
| Hs.235782 | solute carrier organic anion transporter family, member 4A1 | | |
| Hs.495710 | glycoprotein M6B | |  |
| Hs.497253 | chromosome 14 open reading frame 101 | | |
| Hs.191540 | Cohen syndrome 1 | |  |
| Hs.517080 | solute carrier family 35, member C2 | | |
| Hs.310453 | transmembrane 4 superfamily member 14 | | |
|  |  |  |  |
| **DNA PACKAGING** | | **Count: 7** | **P Value = 7.46E-12** |
| Assession No. | Description |  |  |
| Hs.132854 | histone 1, H3f | |  |
| Hs.307924 | male-specific lethal 3-like 1 (Drosophila) | | |
| Hs.182137 | histone 1, H2bo | |  |
| Hs.75307 | H1 histone family, member X | | |
| Hs.534319 | histone 1, H2ad | |  |
| Hs.143080 | histone 4, H4 | |  |
| Hs.121017 | histone 1, H2ab | |  |
|  |  |  |  |
| **MRNA SPLICING** | | **Count: 6** | **P Value = 5.67E-10** |
| Assession No. | Description |  |  |
| Hs.528007 | U2 (RNU2) small nuclear RNA auxiliary factor 2 | | |
| Hs.533736 | RNA binding motif protein 7 | | |
| Hs.175955 | splicing factor YT521-B | |  |
| Hs.6891 | splicing factor, arginine/serine-rich 6 | | |
| Hs.161181 | PRP18 pre-mRNA processing factor 18 homolog | | |
| Hs.149991 | THO complex 2 | |  |
|  |  |  |  |
| **KELCH REPEAT** | | **Count: 5** | **P Value = 1.17E-9** |
| Assession No. | Description |  |  |
| Hs.112569 | giant axonal neuropathy (gigaxonin) | | |
| Hs.508201 | kelch-like 1 (Drosophila) | |  |
| Hs.78788 | leucine-zipper-like transcription regulator 1 | | |
| Hs.440695 | kelch repeat and BTB (POZ) domain containing 4 | | |
| Hs.104925 | ectodermal-neural cortex (with BTB-like domain) | | |
|  |  |  |  |
| **TUMOR ANTIGEN** | | **Count: 5** | **P Value = 2.15E-6** |
| Assession No. | Description |  |  |
| Hs.522665 | melanoma antigen, family D, 2 | | |
| Hs.417816 | melanoma antigen, family A, 3 | | |
| Hs.417816 | melanoma antigen, family A, 6 | | |
| Hs.522803 | melanoma antigen, family A, 8 | | |
| Hs.113290 | melanoma antigen, family B, 3 | | |
|  |  |  |  |
| **GTPASE ACTIVITY** | | **Count: 5** | **P Value = 7.88E-9** |
| Assession No. | Description |  |  |
| Hs.111554 | ADP-ribosylation factor-like 7 | | |
| Hs.194695 | ras homolog gene family, member I | | |
| Hs.245540 | ADP-ribosylation factor-like 4A | | |
| Hs.372616 | ADP-ribosylation factor-like 1 | | |
| Hs.40758 | RAB30, member RAS oncogene family | | |
|  |  |  |  |
| **MONOOXYGENASE ACTIVITY** | | **Count: 5** | **P Value = 1.64E-9** |
| Assession No. | Description |  |  |
| Hs.250615 | cytochrome P450, family 2, subfamily A, polypeptide 7 | | |
| Hs.1361 | cytochrome P450, family 1, subfamily A, polypeptide 2 | | |
| Hs.187393 | cytochrome P450, family 4, subfamily F, polypeptide 11 | | |
| Hs.282624 | cytochrome P450, family 2, subfamily C, polypeptide 9 | | |
| Hs.447793 | cytochrome P450, family 8, subfamily B, polypeptide 1 | | |
